# Molecular Dynamics Insights into Cyrene’s Vapor–Liquid Equilibria and Transport Properties

**DOI:** 10.1021/acs.jpcb.4c08254

**Published:** 2025-01-31

**Authors:** Callum Donaldson, Carmelo Herdes

**Affiliations:** Department of Chemical Engineering, University of Bath, Bath Ba2 7ay, United Kingdom

## Abstract

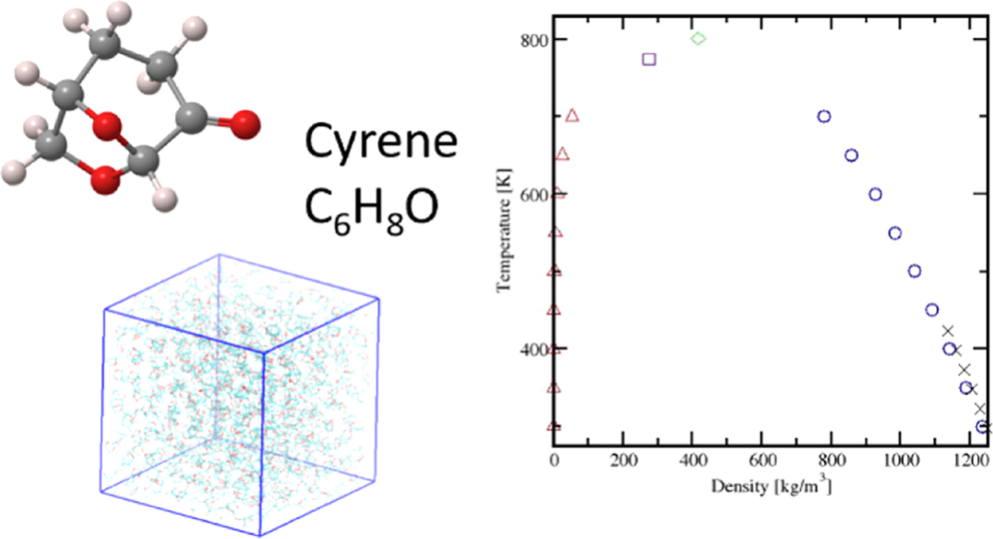

Since its inception in 2014, Cyrene has emerged as a promising biobased solvent derived from renewable cellulose waste, offering a sustainable alternative to conventional toxic solvents. However, experimental data on its thermodynamic and transport properties remain scarce. This study addresses this critical gap by employing state-of-the-art molecular dynamics simulations. The results provide novel data on Cyrene’s phase behavior and fluid dynamics over a wide temperature range (300–700 K) and pressure conditions, including the prediction of critical properties (801 K, 81.04 bar, and 415.389 kg/m^3^). By leveraging advanced computational techniques, this research elucidates Cyrene’s density, diffusion coefficients, and viscosity, with accuracy validated against experimental data where available. These findings enhance our theoretical understanding of Cyrene, supporting its adoption in industrial applications and contributing to the broader agenda of green chemistry. Future work will extend these models to study solvent mixtures and coarse-grained representations, driving further innovation in sustainable solvent design.

## Introduction

Green chemistry focuses on designing chemical products and processes that minimize or eliminate the use and generation of hazardous substances throughout their entire lifecycle, from design and manufacture to use and disposal. It aims to reduce pollution at its source by addressing the hazards associated with chemical feedstocks, reagents, solvents, and products.^1^

Selecting solvents for industrial applications is a technical and environmental consideration. Due to their toxicity and volatility, traditional organic solvents such as toluene, chloroform, dichloromethane, and acetonitrile often pose significant health and environmental risks. In contrast, green solvents offer a solution to these issues.^2−4^

Understanding a solvent’s thermodynamic and transport properties is crucial across various fields due to their significant influence on numerous processes and products. In chemical reactions, these properties affect reaction rates, equilibrium constants, and product distribution, with knowledge of heat capacity, enthalpy, and entropy aiding in understanding reaction energetics.^5^ Solubility and dissolution are governed by temperature, pressure, and solvent–solute interactions, all of which hinge on thermodynamic properties, making them essential in processes like extraction and crystallization. Moreover, thermodynamic properties are central to phase equilibria, impacting vapor–liquid, liquid–liquid, and solid–liquid equilibria, which are critical for designing separation processes and predicting phase transitions. Transport properties are fundamental to mass, momentum, and energy transfer in processes such as filtration, distillation, and heat transfer operations. These properties are also vital for ensuring process safety and minimizing environmental impact, as they play a key role in the design of safe operating conditions, thereby ensuring the safety of the entire process.

Additionally, solvent properties such as polarity and dielectric constant affect material stability and compatibility, influencing the selection of suitable materials for containment and processing. In the pharmaceuticals, food, and electronics industries, controlling solvent properties is not just essential, but it is a key factor in maintaining product quality. Factors such as purity and viscosity, which are directly influenced by solvent properties, play a crucial role in the final product. Thus, a comprehensive understanding of the thermodynamic and transport properties of solvents is not just important but indispensable for efficient process design, optimization, and control across a wide range of applications.

**Figure 1:**
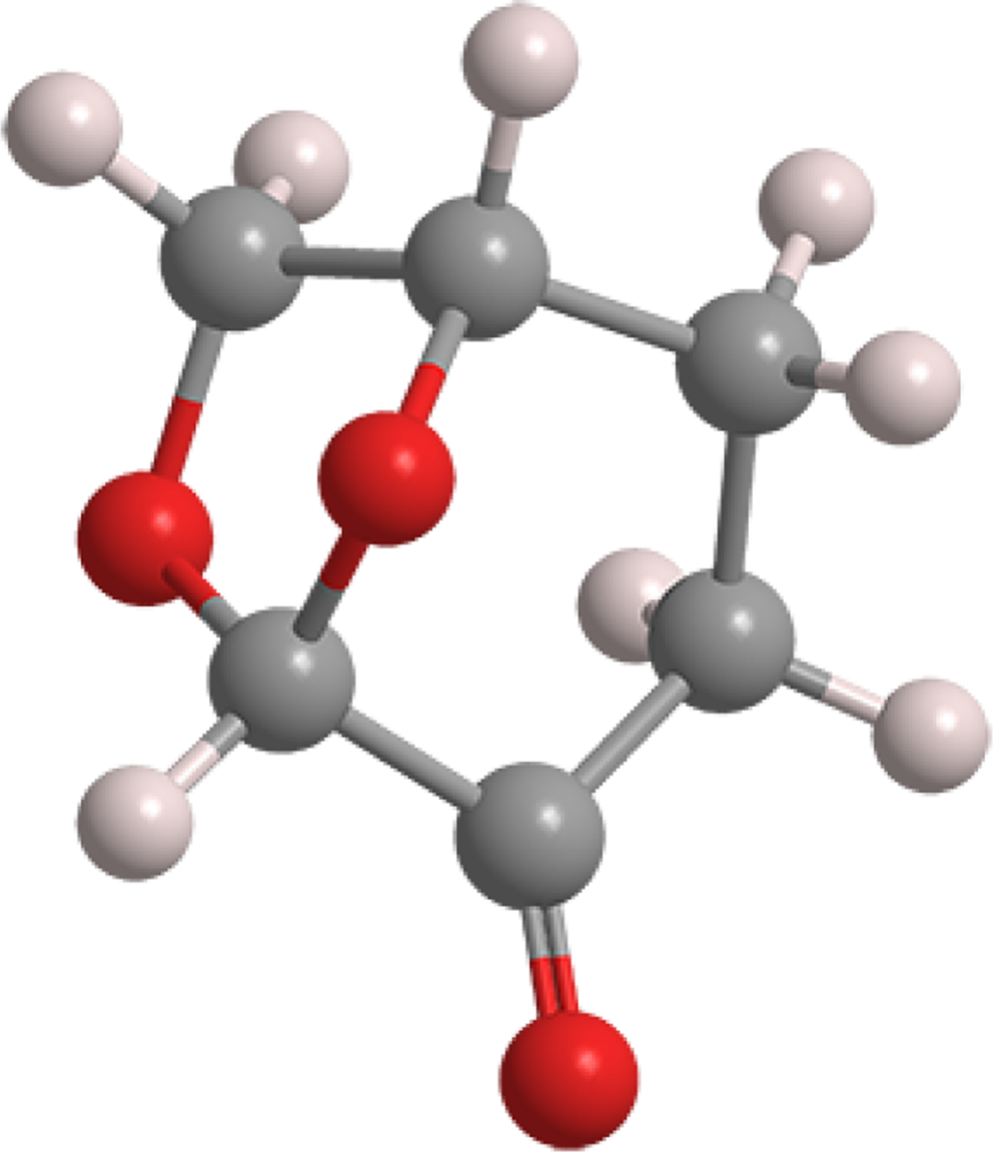
Atomistic representation of the Cyrene molecule. Atom color coding: oxygen: red, carbon: dark gray, and hydrogen: light gray.

Dihydrolevoglucosone (C_6_H_8_O_3_, IUPAC name: (1*S*,5*R*)-6,8-dioxabicyclo[3.2.1]octan-4-one, CAS number: 53716–82–8, trade name: Cyrene) is a bicyclic acetal, dipolar aprotic molecule (Figure 1), developed by the Circa Group in partnership with Professor James Clark, PhD, at the University of York’s Green Chemistry Center of Excellence (GCCE) in 2014.^6^ An indirect connection can be drawn between Cyrene’s role as a sustainable solvent and its name, inspired by the Greek nymph Cyrene, daughter of the river god Peneus, with nymphs symbolizing strength, purity, and harmony with nature.

At room temperature, Cyrene is a colorless to pale yellow liquid with a boiling point of 500 K and a higher density compared to other polar aprotic solvents. Due to the cyclic structure of Cyrene and the arrangement of the acetal and ketone functional groups, Cyrene is highly dipolar, with polarity parameters comparable to NMP (NMP: 0.9 and Cyrene: 0.93).^7^ As with common acetals, it is stable under basic conditions; however, it is susceptible to degradation in the presence of nucleophiles (attack the carbonyl group) and bases (deprotonation of the ketone).^3^

Cyrene is produced through a two-step synthesis from renewable cellulose waste, utilizing an energy-efficient manufacturing process that releases water as a byproduct.^8^ Cyrene has been synthesized from several different biomass starting materials under a variety of conditions, but most of these processes initially form levoglucosenone (LGO). A few examples of these different starting biomass materials are larch log, bagasse, poplar wood, corn cob, crude waste softwood, and bilberry press cake. After the pyrolytic decomposition of cellulose, a reduction of LGO through hydrogenation using palladium-based catalysts such as Pd/Al_2_O_3_ and Pd/C takes place. Additionally, rhodium and zirconia-supported catalysts have also been employed.^3^

The Circa Group has developed a large-scale method for producing Cyrene from cellulose through levoglucosenone (LGO) using the Furacell process, which operates at a capacity of 50 tons of LGO per year. This process adheres to green chemistry principles, focusing on safety, renewable feedstocks, waste minimization, and energy efficiency. The process begins by swelling sawdust with a sulfolane solvent, combined with 2–3% phosphoric acid, to convert cellulose into LGO at 730.15 K. Intermediates such as 5-hydroxymethyl furfural (HMF) and levulinic acid also form during this reaction. The catalyst is activated at 423.15–443.15 K, and the primary reaction proceeds for 5 min, achieving a 40% theoretical yield of LGO along with minor byproducts. The resulting biochar is recycled to fuel the system, which supports energy sustainability. Purification is achieved through fractional distillation, producing LGO with over 90% purity. Cyrene is then synthesized via LGO hydrogenation using a 0.5 mol % palladium catalyst (Pd/C) in ethyl acetate (3:1 molar ratio).^9^

**Figure 2:**
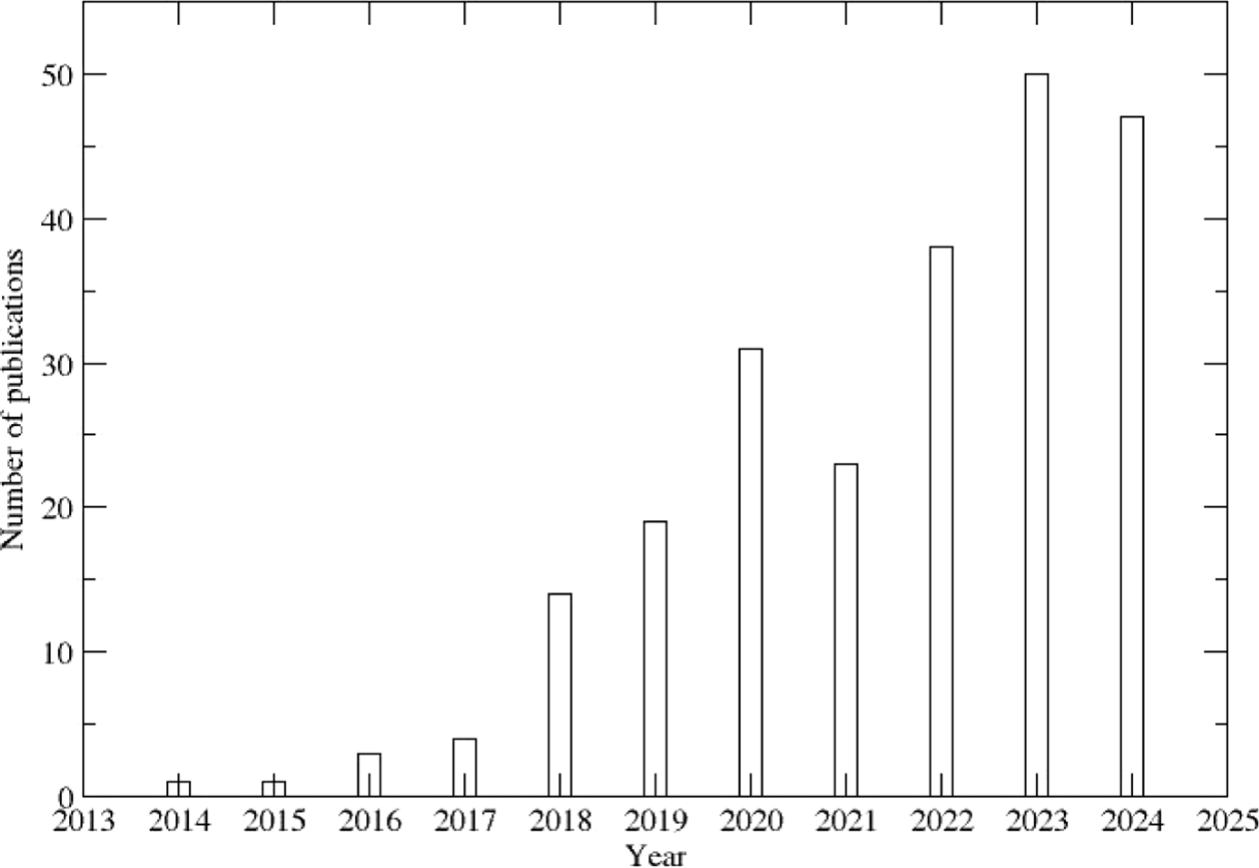
Number of publications per year on Cyrene.

The growing research interest in Cyrene is depicted in Figure 2 as the number of publications per year from 2014 to date. As can be seen, the trend of publications has increased since Cyrene’s inception.

However, to the best of our knowledge, studies involving the use of molecular dynamics (MD) simulations are limited to just four papers. Among these, both Mohan et al.^10^ and Wang et al.^11^ focus on Cyrene’s role as a sustainable solvent for biomass pretreatment, employing both experimental and computational methods to elucidate its impact on lignin. Cytarska et al.’s work delves into Cyrene’s potential as a tyrosinase inhibitor,^12^ while Yin et al. discuss its application within an innovative biorefinery process.^13^

Data on Cyrene’s vapor–liquid equilibria and transport properties are scarce. Available databases show a gap in material property data; while they contain some information regarding properties such as boiling point,^14^ liquid density, and vapor pressure,^15^ they lack the description of the critical point, diffusivity, and viscosity. The absence of material property data for Cyrene has spurred our exploration of MD simulations to predict its vapor–liquid equilibria (VLE), critical point, and transport properties.

In this study, we employ MD simulations using full atomistic models, specifically the groningen molecular simulation (GROMOS)^16^ and all-atom optimized potentials for liquid simulations (AA-OPLS) force fields,^17^ to predict Cyrene’s properties under varying conditions. MD offers significant advantages over traditional experimental methods: it is cost-effective, eliminating the need for chemicals and laboratory setups; it provides faster and more efficient results; it offers detailed molecular insights that are often challenging to obtain experimentally; and it ensures high reproducibility through controlled variables. By complementing experimental data with theoretical support, MD simulations serve as a powerful tool for exploring molecular behavior at a fraction of the cost and time required for physical experiments.

## Methodology and Simulation Details

Parameters in GROMOS are derived from experimental data and quantum mechanical (QM) calculations. They are optimized to reproduce the thermodynamic properties of liquids and other observables. GROMOS is praised for its robustness and extensive validation against experimental data, making it reliable for a variety of biomolecular simulations.

The AA-OPLS force field was developed to provide a reliable and accurate representation of molecular interactions in liquid systems. AA-OPLS is particularly favored in the simulation of organic and biomolecular systems due to its balance of computational efficiency and accuracy.

While QM charge calculations may enhance the accuracy for pure substances, we chose to retain the OPLS charges to ensure consistency with future work focused on Cyrene mixtures with water and acids. This approach leverages the transferable properties of the published force field.

**Figure 3:**
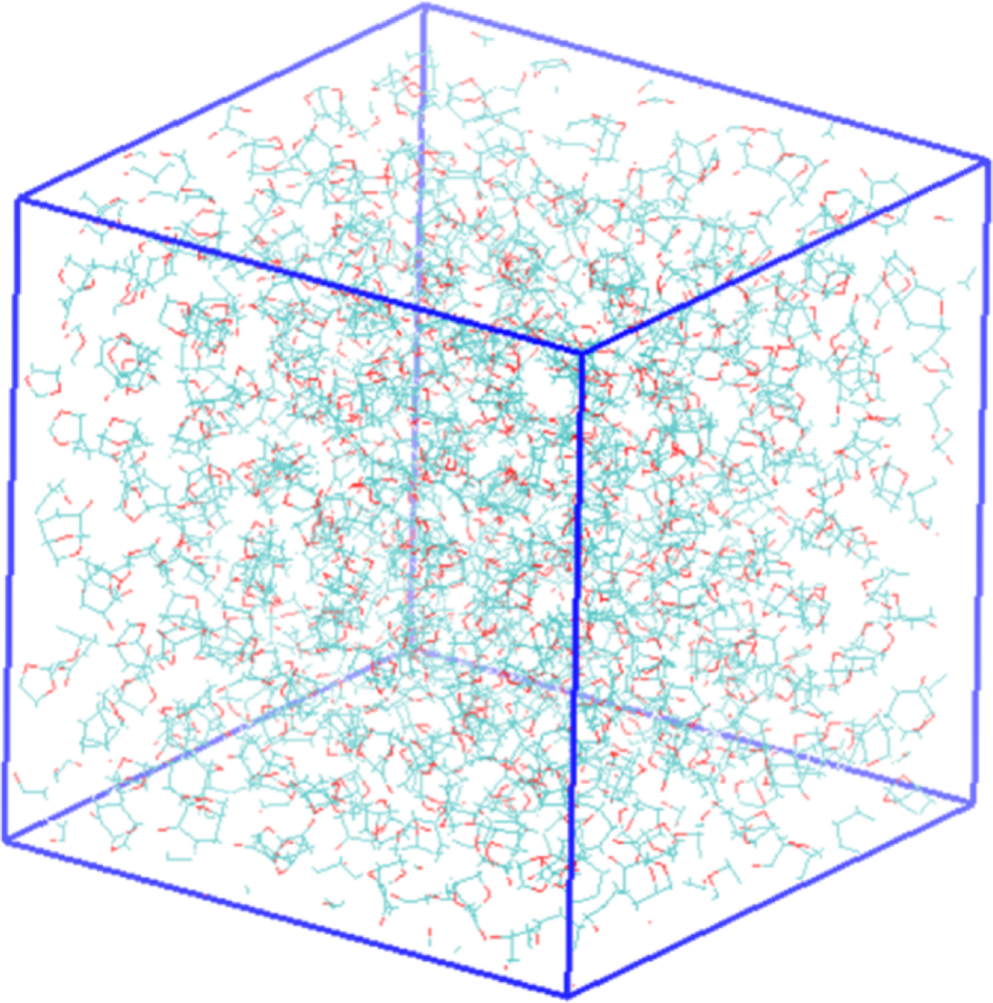
Simulation box with 500 molecules of Cyrene.

A simulation box containing 500 molecules of Cyrene was created for density calculations (Figure 3). After energy minimization, the canonical, NVT, and the isobaric–isothermal, NPT, ensembles were alternated to predict Cyrene’s liquid density over a temperature range of 293 K to 423 K. In both ensembles, simulations were solved with a time step (dt) of 0.001 ps, for 2 and 10 ns equilibration and production time, respectively. The Nose-Hoover thermostat and Parrinello–Rahman barostat were employed for temperature and pressure coupling, respectively. Complete simulation inputs can be found in the Supporting Information accompanying this work.

**Figure 4:**
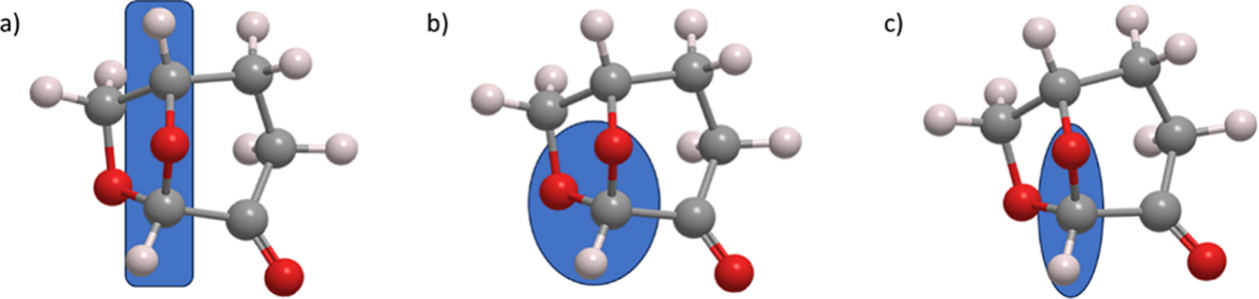
Three studied AA-OPLS models: A) ether, B) acetal, and C) acetal-like.

Three Cyrene models were studied using AA-OPLS parameters based on the selected charge distributions within the molecule (Figure 4).

Simulations were conducted for all four models to predict Cyrene’s density over the same temperature range. These simulations were compared against the experimental densities reported by Jeřábek et al.;^18^ see simulation results for details. The AA-OPLS ether model was found to be the most suitable for density predictions and was used for further simulations, i.e., VLE and transport properties.

The diffusion coefficient was predicted from NVT simulations via GROMAC’s postprocessing function to calculate the mean square displacement (MSD) via the Einstein relation.^19^

Cyrene’s viscosity was predicted via nonequilibrium molecular dynamics (NEMD) simulations in the NVT ensemble, with accelerations ranging from 0.175 to 0.5 nm/ps^2^. Intermediate results and extrapolation to zero acceleration details are given in the Supporting Information file.

For VLE NVT calculations, the number of Cyrene molecules was tripled in a single slab with liquid density, and the box was elongated in the *z*-axis to allow enough room for the vapor phase to develop at various temperatures (300–700 K). The straightforward scaling law method^14^ was used to predict Cyrene’s critical point.

## Results and Discussion

**Figure 5:**
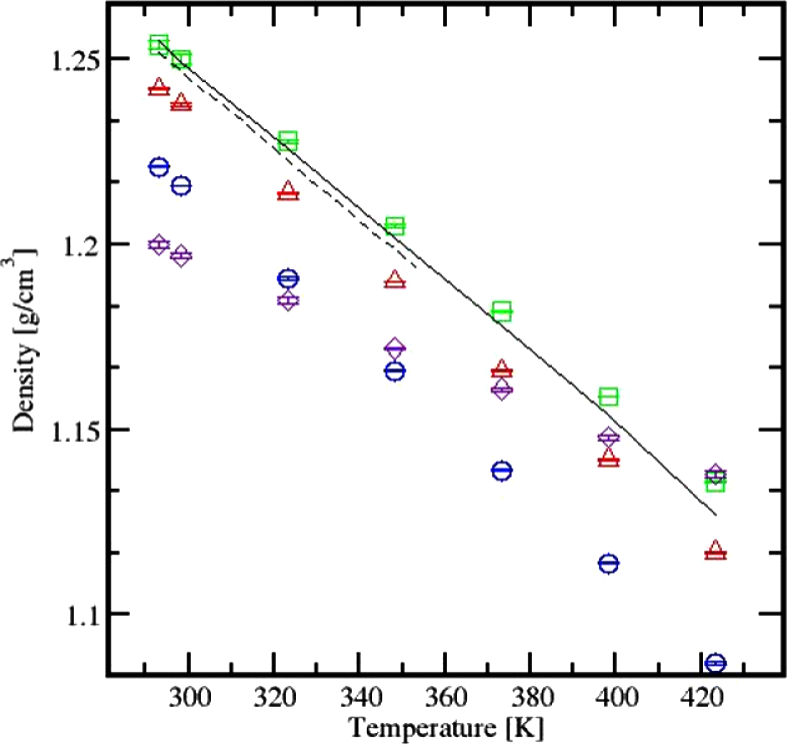
Experimental versus simulated densities. Experimental: Baird et al., dash line; Jerebek et al., continuous line. Simulation: GROMOS ◊, AA-OPLS acetal-like ○, AA-OPLS acetal Δ, and AA-OPLS ether □.

The density of Cyrene was predicted as a function of temperature for all models. Figure 5 showcases the expected linear decreasing trend of the liquid density as the temperature increases.

The GROMOS model was found to predict Cyrene’s density with an average 3.36% deviation from experimental values. In contrast, the AA-OPLS model produced enhanced predictions: 2.59% for the acetal-like model, 0.74% for the acetal one, and 0.32% for the ether model. The difference between the three AA-OPLS models is attributed to the charges allocated to each atom within the molecule, depending on the selected chemical nature. The four models differ primarily in their charge distributions, which are summarized in Table S9. These charges were derived based on the off-the-shelf ATB (GROMOS)^20^ and OPLS^17^ force fields, ensuring consistency with their force field parameterization procedures. The AA-OPLS ether model was chosen for subsequent simulations.

The excellent density predictions obtained with our best model give confidence in the proposed parameters and the suitability of the OPLS force field to describe cyclic structures, such as those in Cyrene. This confidence is supported by extensive prior validation of OPLS for similar structural motifs, including cyclic acetals and ethers.^17^ While additional validation against structurally related molecules would further support our findings, such data are currently unavailable. Future studies will seek to address this gap by obtaining experimental data for related systems.

These density predictions have significant implications for process design and optimization. Accurate density values are crucial in determining volumes, flow rates, and overall process efficiency in industrial settings. Additionally, understanding Cyrene’s density across a range of temperatures aids in optimizing reaction kinetics, ensuring proper storage conditions, and implementing effective safety measures.

**Figure 6:**
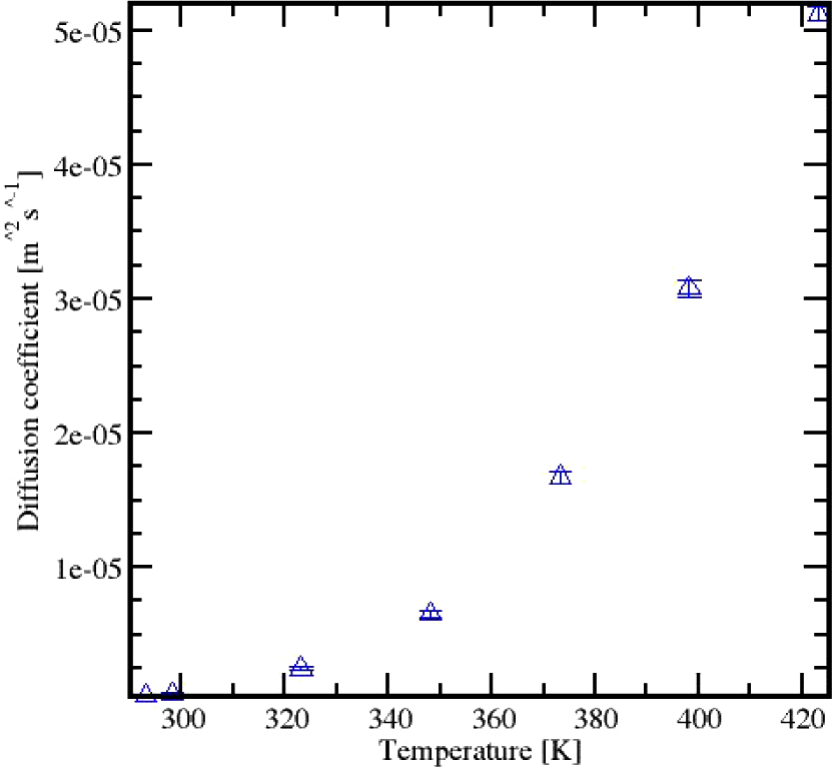
Cyrene diffusion coefficient prediction.

We also investigated the diffusion coefficients of Cyrene as a function of temperature. Across the temperature range studied, the diffusion coefficients remained consistently low, approximately on the order of 10^–7^ m^2^/s. An increase in the diffusion coefficient was observed with rising temperature. Specifically, the predicted diffusion coefficient increased from 4.453 × 10^–7^ m^2^/s at 293 K to 5.124 × 10^–7^ m^2^/s at 423 K. This change represents a significant increase, but it is still indicative of overall low molecular mobility. Figure 6 illustrates these results, highlighting the relationship between temperature and diffusion coefficient.

The predicted diffusivity values were corrected for system size effects following the methodologies described elsewhere.^21^ The corrections account for finite-size artifacts in molecular dynamics simulations and were applied to data for which viscosity and box length information were available. The adjustments resulted in minimal changes, with differences observed only at the fifth decimal place, indicating that system size effects had a negligible impact on the overall trends. These findings are consistent with the expectation that corrections for the remaining temperatures would similarly produce only minor adjustments and that the corrected values align well with the original predictions.

Understanding these diffusion characteristics is crucial for applications requiring precise control over molecular mobility. Low diffusion coefficients can lead to sluggish reaction rates and reduced efficiency in solvent extraction processes. Recognizing these limitations emphasizes the need to optimize processes in various industrial applications. We could not validate our predictions as experimental data on Cyrene’s diffusion coefficient were not found.

**Figure 7:**
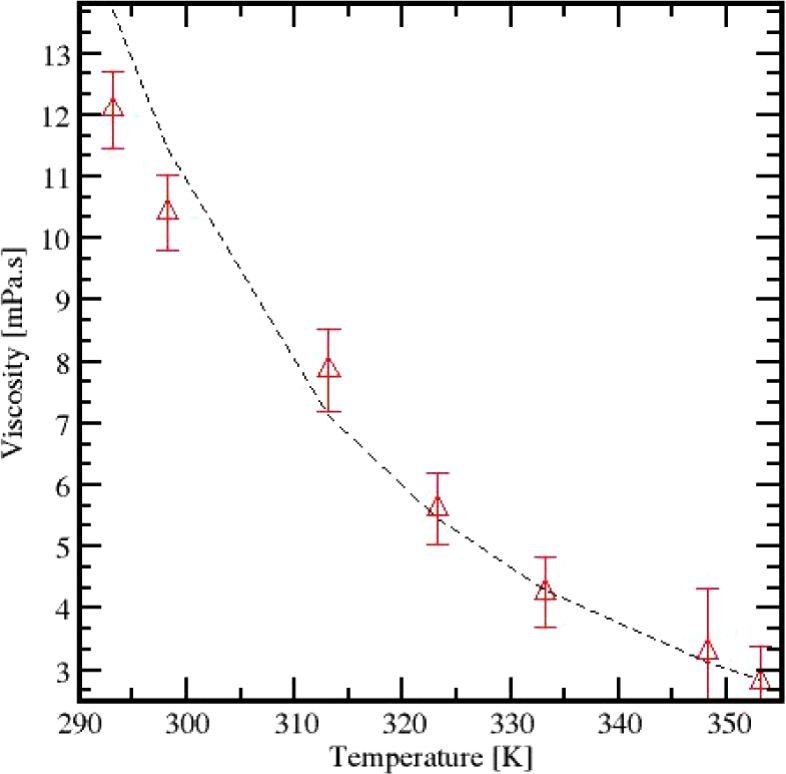
Comparison of Cyrene viscosity predictions vs experimentally obtained values of Cyrene over a range of temperatures.

The viscosity of Cyrene was predicted across a range of temperatures (293–353 K). This is illustrated in Figure 7, which shows a smooth, continuous decline in viscosity with rising temperature, fitting an almost exponential trendline.

The observed changes in viscosity, with an average error rate of 0.56%, indicate reassurance in the transport properties prediction. Accurate viscosity values are crucial for a wide range of applications, including chemical processing, materials science, polymer processing, food production, and pharmaceutical manufacturing. Additionally, optimal viscosity control can enhance efficiency and reduce environmental impact by minimizing resource wastage.

While additional NEMD simulations could reduce error bars and improve the fits presented in the Supporting Information, the current viscosity predictions as a function of temperature (Figure 7) exhibit a strong and consistent trend. This alignment with expected physical behavior validates the reliability of the methodology used. As such, we consider the current data set adequate for characterizing Cyrene’s viscosity trends and observing its temperature dependence.

**Figure 8:**
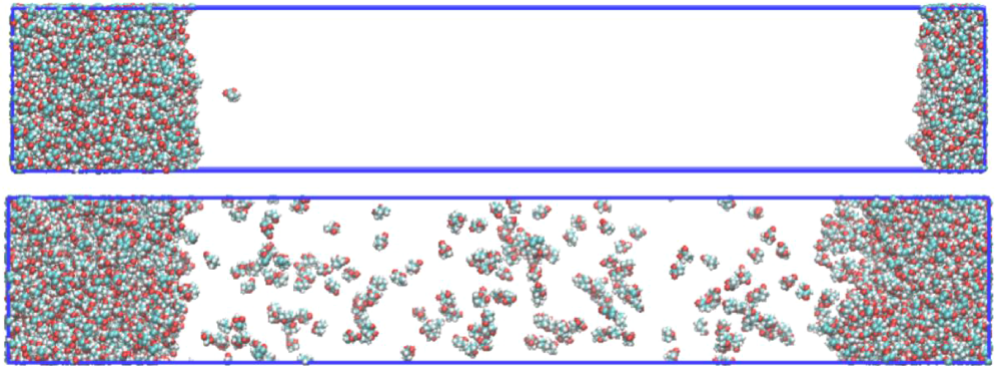
Final configurations of the simulation box at different temperatures. Top: −300 K; bottom: 700 K.

Figure 8 depicts the development of the Cyrene vapor phase at the lowest and highest temperatures investigated here.

**Figure 9:**
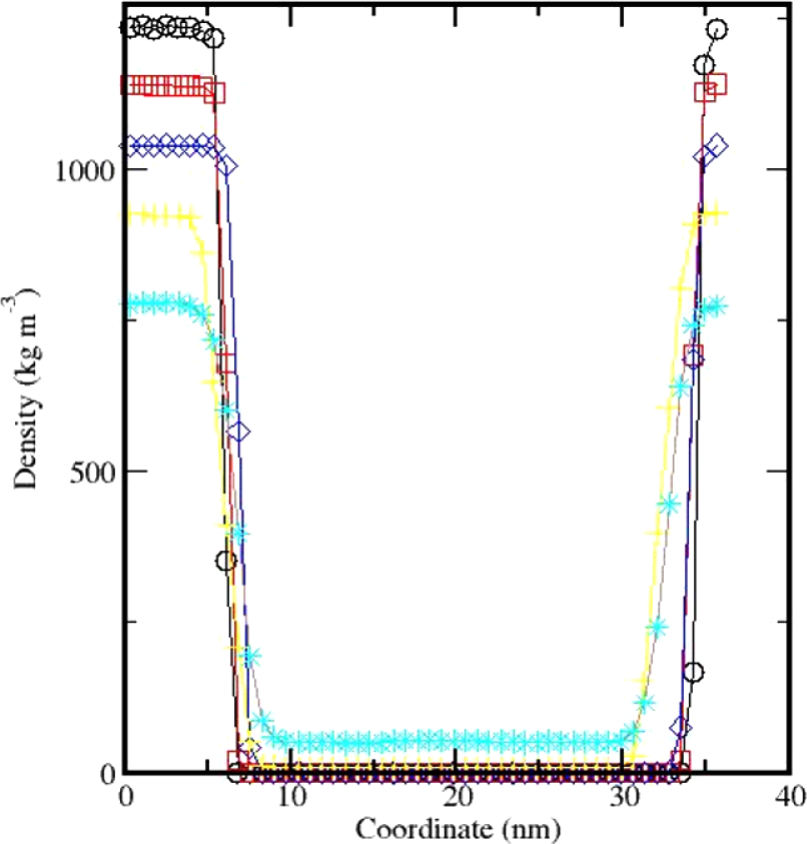
Density profile of Cyrene at 300 K ○, 400 K □, 500 K ◊, 600 K +, and 700 K ∗; the lines are a guide to the eye.

**Figure 10:**
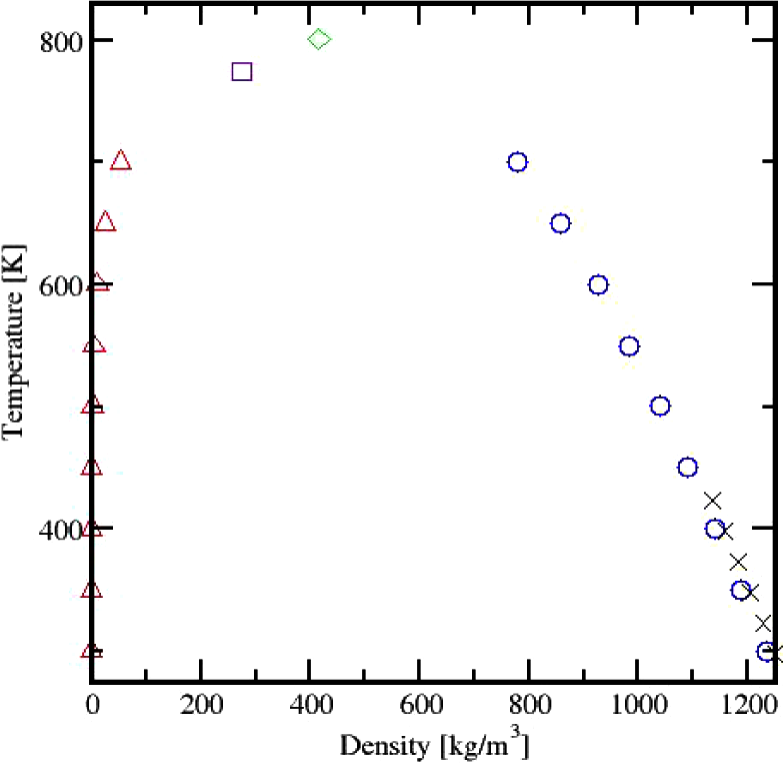
Vapor–liquid coexistence curve for Cyrene. Vapor Δ, liquid ○, density from NPT simulation × , model critical point from scaling law ◊, and critical point from ref (18) □.

By exploring the *z*-directional density profiles of Cyrene at different temperatures, the vapor and liquid density in equilibrium can be obtained, see Figure 9. As the temperature increases, the liquid density gradually decreases, and the corresponding vapor density is shown to gradually increase. From this data, we were able to obtain averages of the density profiles for the vapor and liquid regions. These average liquid and vapor densities were then plotted against temperature to produce a vapor–liquid coexistence curve (Figure 10). From the simulation results at different temperatures, the critical temperature was determined using the scaling law method (eq 1). These laws describe how the properties of a system behave near the critical point, specifically relating the densities to the reduced temperature, .
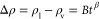
1

Where *T*_c_ is the critical temperature and β is the critical exponent. The data were linearized by plotting log Δρ against log *t* to produce a straight line with the slope β (see the Supporting Information file). As such, 801 K is the critical temperature, *T*_c_, of our proposed model with an *R*^2^ value of 0.9996, a critical exponent, β, of 0.326, and a critical density of 415.389 kg/m^3^. The van der Waals equation of state was solved for a critical pressure of 81.04 bar. With the calculated VLE and critical point of Cyrene, we predict its acentric factor, ω, to be −0.234. However, Cyrene’s very low vapor pressure^18^ poses challenges for accurately predicting its vapor pressure, leading to inaccuracies in the acentric factor calculation. The scaling law used for critical property predictions, while effective, inherently simplifies the complex behavior near critical points, potentially leading to inaccuracies that experimental validation can resolve but are out of the scope of this work.

The critical point estimates provided in this study are based on extrapolated vapor and liquid densities using the scaling law method. While the predicted critical temperature of 801 K aligns with expected trends, distinguishing phase densities near 750 K proved challenging due to phase convergence. This limitation underscores the complexity of simulating near-critical behavior and highlights the need for experimental validation. The predicted critical properties will support future coarse-grained parametrization efforts using the corresponding states approach.^22^ For reproducibility, simulations at high temperatures are included in the Supporting Information.

The predicted VLE behavior of Cyrene underscores its potential for industrial applications requiring precise phase control. The predicted phase envelope indicates Cyrene’s wide operating range, as well as its robustness in challenging operational environments, particularly toward applications with supercritical fluids such as cleaning, extraction, or reactions, which is of particular importance with regards to the absence of experimental data.

The direct coexistence method, employed for VLE predictions in this study, provides a straightforward framework, leveraging the ergodic hypothesis to observe vapor and liquid phases in equilibrium under identical simulation conditions. While this method is less efficient than Monte Carlo simulations, which excel in computational precision and free-energy-based phase equilibrium calculations, it remains advantageous for capturing molecular interactions in complex systems like Cyrene. This is particularly valuable, given the scarcity of experimental data for such systems.

Furthermore, Monte Carlo methods are inherently unsuitable for obtaining transport properties, such as diffusion and viscosity. Since this study focuses on both equilibrium and transport properties, the direct coexistence method coupled with molecular dynamics provides a comprehensive and indispensable approach.

Future work will focus on utilizing Cyrene as a green solvent in prepolymerization mixtures for molecularly imprinted polymers (MIPs), where molecular dynamics simulations are essential for studying species diffusion.^23^ Additionally, combining Monte Carlo simulations for parametrization with molecular dynamics to exploit the ergodic hypothesis offers a promising path for achieving greater accuracy and efficiency in VLE predictions.

Finally, the four molecular models studied here offer different calculated dipole moments, reflecting variations in their charge distributions, with the GROMOS model at 5.2 D, acetal at 1.35 D, acetal-like at 4.24 D, and ether at 5.77 D, all differing from the reported experimental value of 3.4 D.^14^

The dipole moment of molecular models is a critical property influencing intermolecular interactions such as hydrogen bonding, van der Waals forces, and electrostatic interactions, which collectively govern the properties predicted by simulations. For example, properties like the diffusion coefficient and viscosity can be significantly affected by the dipole moment. A higher dipole moment typically corresponds to stronger intermolecular attractions, potentially leading to increased viscosity and reduced diffusion coefficients. Although the dipole moment of the ether model deviates most significantly from Cyrene’s actual dipole moment, it demonstrated remarkable accuracy in predicting density and viscosity over a wide range of temperatures.

## Conclusion

We have presented a comprehensive computational characterization of Cyrene, a sustainable biobased solvent, addressing significant knowledge gaps in its thermodynamic and transport properties. By employing molecular dynamics simulations with robust GROMOS and AA-OPLS force fields, we have predicted Cyrene’s density, viscosity, and diffusion coefficients across a broad temperature range with high accuracy. Additionally, the study provides critical property predictions using scaling laws, advancing our understanding of Cyrene’s phase behavior under diverse conditions.

The findings underline Cyrene’s potential for industrial applications requiring robust, sustainable solvents, particularly in processes involving supercritical fluids, solvent recovery, and green manufacturing. The ether-based AA-OPLS model demonstrated exceptional performance, offering a reliable basis for future simulations. However, the study also highlights the need for experimental validation of diffusion coefficients and vapor pressure, as well as refinements in force field parameters to improve the prediction of dipolar interactions.

Looking forward, future research will develop coarse-grained models to extend these insights to complex solvent systems and larger temporal scales. This work not only contributes to the fundamental understanding of Cyrene but also reinforces its role as a key enabler in achieving greener, more sustainable industrial processes, aligned with the principles of green chemistry.

## Supporting Information Available

The Cyrene GROMOS model, Cyrene AA-OPLS acetal model, Cyrene AA-OPLS acetal-like model, and Cyrene AA-OPLS ether model; energy minimization, NVT simulation, and NPT simulation; molecular structure of Cyrene; summary of oxygen partial charges across different models; viscosity extrapolation vs acceleration data; scaling law fit for critical property predictions; dipole moment methodology and computation details; corrected diffusion methodology and computation details (PDF)
