# Supplementary material for: Molecular Dynamics Insights into Cyrene’s Vapor–Liquid Equilibria and Transport Properties
Source: J Phys Chem B. 2025 Jan 31;129(6):1811–7. doi: 10.1021/acs.jpcb.4c08254 (PMC11831665; doi:10.1021/acs.jpcb.4c08254)
Supplement: Supplementary file 1 — jp4c08254_si_001.pdf [file jp4c08254_si_001.pdf]

Molecular Dynamics Insights into Cyrene's Vapour-Liquid Equilibria and Transport Properties

Callum Donaldson, Carmelo Herdes*

Department of Chemical Engineering, University of Bath, Claverton Down, Bath, Somerset BA2 7AY, United Kingdom

*Corresponding author email: cehm21@bath.ac.uk

Supporting information

Overview: This supplementary document provides detailed methodologies, parameter files, and supporting data to complement the findings in the main manuscript. The sections are organized to facilitate easy reference for researchers replicating or building upon this work.

Table of Contents

1. **Simulation Parameter Files**
   - SI1: Cyrene GROMOS .itp File
   - SI2: Cyrene AA-OPLS Acetal Model .itp File
   - SI3: Cyrene AA-OPLS Acetal-like Model .itp File
   - SI4: Cyrene AA-OPLS Ether Model .itp File
2. **Molecular Dynamics Parameter Files**
   - SI5: Energy Minimization (em.mdp)
   - SI6: NVT Simulation (NVT.mdp)
   - SI7: NPT Simulation (NPT.mdp)
3. **Molecular Structure File**
   - SI8: Cyrene .gro File
4. **Comparison of charge differences between models**
   - SI9: Table summary of oxygen partial charges across different models
5. **Results Supporting Figures**
   - SI10: Viscosity Extrapolation vs Acceleration Data
   - SI11: Scaling Law Fit for Critical Property Predictions

6. **Dipole Moment Calculation**
   - SI12: Methodology and Computation Details
7. **Corrected diffusion for system size effects**
   - SI13: Methodology and Computation Details

1 Simulation Parameter Files:

- S1-S4: Includes .itp files generated for GROMOS and AA-OPLS force fields. These files define atom types, charges, bonds, and angles, enabling accurate molecular simulations of Cyrene.
  - S1: GROMOS-based file from ATB with balanced charges.
  - S2-S4: AA-OPLS models (acetal, acetal-like, ether) generated using mktop, with variations in charge distributions to investigate model sensitivity.

SI1: Cyrene itp file (molid: 714277) generated by Automated Topology Builder (ATB) and Repository (https://atb.uq.edu.au/) using GROMOS forcefield

```
[ atoms ]
;  nr  type  resnr  resid  atom  cgnr  charge    mass
   1 OEOpt    1    LDMS    O3    1   -0.500  15.9994
   2  CPos    1    LDMS    C6    2    0.372  12.0110
   3  CPos    1    LDMS    C3    3    0.402  12.0110
   4    HC    1    LDMS    H4    4    0.050   1.0080
   5    OE    1    LDMS    O1    5   -0.425  15.9994
   6     C    1    LDMS    C1    6    0.164  12.0110
   7    HC    1    LDMS    H1    7    0.086   1.0080
   8  CPos    1    LDMS    C4    8    0.040  12.0110
   9    OE    1    LDMS    O2    9   -0.440  15.9994
  10    HC    1    LDMS    H5   10    0.085   1.0080
  11    HC    1    LDMS    H6   11    0.085   1.0080
  12     C    1    LDMS    C2   12   -0.191  12.0110
  13    HC    1    LDMS    H2   13    0.094   1.0080
  14    HC    1    LDMS    H3   14    0.067   1.0080
  15     C    1    LDMS    C5   15    0.010  12.0110
  16    HC    1    LDMS    H7   16    0.042   1.0080
  17    HC    1    LDMS    H8   17    0.059   1.0080
; total charge of the molecule:   0.000
[ bonds ]
;  ai   aj  funct   c0         c1
   1    2    2   0.1220   2.2843e+07
   2    3    2   0.1530   7.1500e+06
   2   15    2   0.1520   5.4300e+06
   3    4    2   0.1090   1.2300e+07
   3    5    2   0.1410   6.5389e+06
   3    9    2   0.1430   8.1800e+06
```

```
   5    6    2   0.1450   5.2319e+06
   6    7    2   0.1090   1.2300e+07
   6    8    2   0.1530   7.1500e+06
   6   12    2   0.1530   7.1500e+06
   8    9    2   0.1450   5.2319e+06
   8   10    2   0.1090   1.2300e+07
   8   11    2   0.1090   1.2300e+07
  12   13    2   0.1090   1.2300e+07
  12   14    2   0.1090   1.2300e+07
  12   15    2   0.1540   4.0057e+06
  15   16    2   0.1090   1.2300e+07
  15   17    2   0.1090   1.2300e+07
```

SI2: Cyrene acetal model .itp file using AA-OPLS forcefield. Modified from GROMOS.itp using mktop

```
[ atoms ]
;  nr  type       resnr  resid  atom  cgnr  charge    mass
    1  opls_281    1      LDM    O3    4    -0.470    15.9994
    2  opls_280    1      LDM    C6    4     0.470    12.011
    3  opls_193    1      LDM    C3    1     0.300    12.011
    4  opls_194    1      LDM    H4    1     0.100    1.008
    5  opls_186    1      LDM    O1    1    -0.400    15.9994
    6  opls_137    1      LDM    C1    3    -0.060    12.011
    7  opls_140    1      LDM    H1    3     0.060    1.008
    8  opls_184    1      LDM    C4    6     0.300    12.011
    9  opls_186    1      LDM    O2    6    -0.400    15.9994
   10  opls_185    1      LDM    H5    6     0.050    1.008
   11  opls_185    1      LDM    H6    6     0.050    1.008
   12  opls_136    1      LDM    C2    2    -0.120    12.011
   13  opls_140    1      LDM    H2    2     0.060    1.008
   14  opls_140    1      LDM    H3    2     0.060    1.008
   15  opls_136    1      LDM    C5    5    -0.120    12.011
   16  opls_140    1      LDM    H7    5     0.060    1.008
   17  opls_140    1      LDM    H8    5     0.060    1.008
; total charge of the molecule:   0.000
[ bonds ]
;  ai   aj  funct   c0        c1
   1    2    2   0.1220   2.2843e+07
   2    3    2   0.1530   7.1500e+06
   2   15    2   0.1520   5.4300e+06
   3    4    2   0.1090   1.2300e+07
   3    5    2   0.1410   6.5389e+06
   3    9    2   0.1430   8.1800e+06
   5    6    2   0.1450   5.2319e+06
   6    7    2   0.1090   1.2300e+07
   6    8    2   0.1530   7.1500e+06
   6   12    2   0.1530   7.1500e+06
   8    9    2   0.1450   5.2319e+06
   8   10    2   0.1090   1.2300e+07
   8   11    2   0.1090   1.2300e+07
```

```
  12   13    2   0.1090   1.2300e+07
  12   14    2   0.1090   1.2300e+07
  12   15    2   0.1540   4.0057e+06
  15   16    2   0.1090   1.2300e+07
  15   17    2   0.1090   1.2300e+07
```

SI3: Cyrene acetal-like model .itp file using AA-OPLS forcefield. Modified from GROMOS.itp using mktop

```
[ atoms ]
;  nr  type       resnr  resid  atom  cgnr  charge    mass
    1  opls_281    1      LDM    O3    4    -0.470     15.9994
    2  opls_280    1      LDM    C6    4     0.470     12.011
    3  opls_193    1      LDM    C3    1     0.300     12.011
    4  opls_194    1      LDM    H4    1     0.100     1.008
    5  opls_186    1      LDM    O1    1    -0.200     15.9994
    6  opls_137    1      LDM    C1    3    -0.060     12.011
    7  opls_140    1      LDM    H1    3     0.060     1.008
    8  opls_136    1      LDM    C4    6    -0.120     12.011
    9  opls_186    1      LDM    O2    1    -0.200     15.9994
   10  opls_140    1      LDM    H5    6     0.060     1.008
   11  opls_140    1      LDM    H6    6     0.060     1.008
   12  opls_136    1      LDM    C2    2    -0.120     12.011
   13  opls_140    1      LDM    H2    2     0.060     1.008
   14  opls_140    1      LDM    H3    2     0.060     1.008
   15  opls_136    1      LDM    C5    5    -0.120     12.011
   16  opls_140    1      LDM    H7    5     0.060     1.008
   17  opls_140    1      LDM    H8    5     0.060     1.008
; total charge of the molecule:   0.000
[ bonds ]
;  ai   aj  funct   c0         c1
    1    2    2   0.1220   2.2843e+07
    2    3    2   0.1530   7.1500e+06
    2   15    2   0.1520   5.4300e+06
    3    4    2   0.1090   1.2300e+07
    3    5    2   0.1410   6.5389e+06
    3    9    2   0.1430   8.1800e+06
    5    6    2   0.1450   5.2319e+06
    6    7    2   0.1090   1.2300e+07
    6    8    2   0.1530   7.1500e+06
    6   12    2   0.1530   7.1500e+06
    8    9    2   0.1450   5.2319e+06
    8   10    2   0.1090   1.2300e+07
    8   11    2   0.1090   1.2300e+07
   12   13    2   0.1090   1.2300e+07
   12   14    2   0.1090   1.2300e+07
   12   15    2   0.1540   4.0057e+06
   15   16    2   0.1090   1.2300e+07
   15   17    2   0.1090   1.2300e+07
```

SI4: Cyrene ether model .itp file using AA-OPLS forcefield. Modified from GROMOS.itp using mktop

```
[ atoms ]
;  nr  type       resnr  resid  atom  cgnr  charge    mass
    1  opls_281    1     CYR    O3     1    -0.470   15.9994
    2  opls_280    1     CYR    C6     1     0.470   12.0110
    3  opls_183    1     CYR    C3     2     0.170   12.0110
    4  opls_185    1     CYR    H4     2     0.080    1.0080
    5  opls_180    1     CYR    O1     2    -0.400   15.9994
    6  opls_183    1     CYR    C1     2     0.170   12.0110
    7  opls_185    1     CYR    H1     2     0.080    1.0080
    8  opls_182    1     CYR    C4     2     0.140   12.0110
    9  opls_180    1     CYR    O2     2    -0.400   15.9994
   10  opls_185    1     CYR    H5     2     0.080    1.0080
   11  opls_185    1     CYR    H6     2     0.080    1.0080
   12  opls_136    1     CYR    C2     3    -0.120   12.0110
   13  opls_140    1     CYR    H2     3     0.060    1.0080
   14  opls_140    1     CYR    H3     3     0.060    1.0080
   15  opls_136    1     CYR    C5     4    -0.120   12.0110
   16  opls_140    1     CYR    H7     4     0.060    1.0080
   17  opls_140    1     CYR    H8     4     0.060    1.0080
; total charge of the molecule:   0.000
[ bonds ]
;  ai   aj  funct   c0        c1
    1    2    2   0.1220   2.2843e+07
    2    3    2   0.1530   7.1500e+06
    2   15    2   0.1520   5.4300e+06
    3    4    2   0.1090   1.2300e+07
    3    5    2   0.1410   6.5389e+06
    3    9    2   0.1430   8.1800e+06
    5    6    2   0.1450   5.2319e+06
    6    7    2   0.1090   1.2300e+07
    6    8    2   0.1530   7.1500e+06
    6   12    2   0.1530   7.1500e+06
    8    9    2   0.1450   5.2319e+06
    8   10    2   0.1090   1.2300e+07
    8   11    2   0.1090   1.2300e+07
   12   13    2   0.1090   1.2300e+07
   12   14    2   0.1090   1.2300e+07
   12   15    2   0.1540   4.0057e+06
   15   16    2   0.1090   1.2300e+07
   15   17    2   0.1090   1.2300e+07
```

2 Molecular Dynamics Parameter Files:

- S5-S7: .mdp files provide settings for energy minimization, canonical (NVT), and isobaric-isothermal (NPT) ensemble simulations. These files

include details such as integrators, temperature and pressure coupling, and cut-off schemes.

SI5: Energy minimisation molecular dynamics parameters (em.mdp)

```
integrator              = steep
nsteps                  = 50000
nstenergy               = 500
nstlog                  = 500
nstxout-compressed      = 1000
cutoff-scheme           = Verlet
coulombtype             = PME
rcoulomb                = 1.0
vdwtype                 = Cut-off
rvdw                    = 1.0
DispCorr                = EnerPres
```

SI6: NVT molecular dynamics parameters (NVT.mdp)

```
; RUN CONTROL PARAMETERS
integrator               = md
; Start time and timestep in ps
tinit                    = 0
dt                       = 0.001
nsteps                   = 2000000
; For exact run continuation or redoing part of a run
init-step                = 0
; mode for center of mass motion removal
comm-mode                = Linear
; number of steps for center of mass motion removal
nstcomm                  = 100
; OUTPUT CONTROL OPTIONS
; Output frequency for coords (x), velocities (v) and forces (f)
nstxout                  = 1000
nstvout                  = 1000
nstfout                  = 1000
; Output frequency for energies to log file and energy file
nstlog                   = 1000
nstcalcenergy            = 1000
nstenergy                = 1000
; Output frequency and precision for .xtc file
nstxout-compressed       = 1000
compressed-x-precision   = 1000
; This selects the subset of atoms for the compressed
; trajectory file. You can select multiple groups. By
; default, all atoms will be written.
compressed-x-grps        =
; Selection of energy groups
energygrps               =
```

```
; NEIGHBORSEARCHING PARAMETERS
; cut-off scheme (Verlet: particle based cut-offs)
cutoff-scheme            = Verlet
; nblist update frequency
nstlist                  = 10
; Periodic boundary conditions: xyz, no, xy
pbc                      = xyz
; Allowed energy error due to the Verlet buffer in kJ/mol/ps per atom,
; a value of -1 means: use rlist
verlet-buffer-tolerance  = 0.005
; nblist cut-off
rlist                    = 1.2
; long-range cut-off for switched potentials
; OPTIONS FOR ELECTROSTATICS AND VDW
; Method for doing electrostatics
coulombtype              = PME
coulomb-modifier         = Potential-shift-Verlet
rcoulomb-switch          = 0
rcoulomb                 = 1.2
; Method for doing Van der Waals
vdwtype                  = Cut-off
vdw-modifier             = Potential-shift-Verlet
; cut-off lengths
rvdw-switch              = 0
rvdw                     = 1.2
; Apply long range dispersion corrections for Energy and Pressure
DispCorr                 = EnerPres
; Extension of the potential lookup tables beyond the cut-off
table-extension          = 1
; Separate tables between energy group pairs
energygrp-table          =
; Spacing for the PME/PPPM FFT grid
fourierspacing           = 0.12
; FFT grid size, when a value is 0 fourierspacing will be used
fourier-nx               = 0
fourier-ny               = 0
fourier-nz               = 0
; EWALD/PME/PPPM parameters
pme-order                = 4
ewald-rtol               = 1e-05
ewald-rtol-lj            = 0.001
lj-pme-comb-rule         = Geometric
ewald-geometry           = 3d
epsilon-surface          = 0
implicit-solvent         = no
; OPTIONS FOR WEAK COUPLING ALGORITHMS
; Temperature coupling
tcoupl                   = Berendsen ;Nose-Hoover
; Groups to couple separately
tc-grps                  = System
; Time constant (ps) and reference temperature (K)
```

```
tau-t                = 1.0
ref-t                = 298.15
; pressure coupling
pcoupl               = No
pcoupltype           = Isotropic
; Time constant (ps), compressibility (1/bar) and reference P (bar)
tau-p                = 1
compressibility      = 4.5e-5
ref-p                = 1.0
; GENERATE VELOCITIES FOR STARTUP RUN
gen-vel              = no
gen-temp             = 298.15
gen-seed             = -1
; OPTIONS FOR BONDS
constraints          = h-bonds
; Type of constraint algorithm
constraint-algorithm = lincs
; Do not constrain the start configuration
continuation         = no
; Use successive overrelaxation to reduce the number of shake iterations
Shake-SOR            = no
; Relative tolerance of shake
shake-tol            = 0.0001
; Highest order in the expansion of the constraint coupling matrix
lincs-order          = 4
; Number of iterations in the final step of LINCS. 1 is fine for
; normal simulations, but use 2 to conserve energy in NVE runs.
; For energy minimization with constraints it should be 4 to 8.
lincs-iter           = 1
; Lincs will write a warning to the stderr if in one step a bond
; rotates over more degrees than
lincs-warnangle      = 30
; Convert harmonic bonds to morse potentials
morse                = no
; Non-equilibrium MD stuff
;acc-grps            =
;accelerate          =
;freezegrps          =
;freezedim           =
;cos-acceleration    = 0
;deform              =
```

SI7: NPT molecular dynamics parameters (NPT.mdp)

```
; RUN CONTROL PARAMETERS
integrator           = md
; Start time and timestep in ps
tinit                = 0
dt                   = 0.001
nsteps               = 10000000
```

```
; For exact run continuation or redoing part of a run
init-step                = 0
; mode for center of mass motion removal
comm-mode                = Linear
; number of steps for center of mass motion removal
nstcomm                  = 100
; OUTPUT CONTROL OPTIONS
; Output frequency for coords (x), velocities (v) and forces (f)
nstxout                  = 1000
nstvout                  = 1000
nstfout                  = 1000
; Output frequency for energies to log file and energy file
nstlog                   = 1000
nstcalcenergy            = 1000
nstenergy                = 1000
; Output frequency and precision for .xtc file
nstxout-compressed       = 1000
compressed-x-precision   = 1000
; This selects the subset of atoms for the compressed
; trajectory file. You can select multiple groups. By
; default, all atoms will be written.
compressed-x-grps        =
; Selection of energy groups
energygrps               =
; NEIGHBORSEARCHING PARAMETERS
; cut-off scheme (Verlet: particle based cut-offs)
cutoff-scheme            = Verlet
; nblist update frequency
nstlist                  = 10
; Periodic boundary conditions: xyz, no, xy
pbc                      = xyz
; Allowed energy error due to the Verlet buffer in kJ/mol/ps per atom,
; a value of -1 means: use rlist
verlet-buffer-tolerance  = 0.005
; nblist cut-off
rlist                    = 1
; long-range cut-off for switched potentials
; OPTIONS FOR ELECTROSTATICS AND VDW
; Method for doing electrostatics
coulombtype              = PME
coulomb-modifier         = Potential-shift-Verlet
rcoulomb-switch          = 0
rcoulomb                 = 1.2
; Method for doing Van der Waals
vdwtype                  = Cut-off
vdw-modifier             = Potential-shift-Verlet
; cut-off lengths
rvdw-switch              = 0
rvdw                     = 1.2
; Apply long range dispersion corrections for Energy and Pressure
DispCorr                 = EnerPres
```

```
; Extension of the potential lookup tables beyond the cut-off
table-extension          = 1
; Separate tables between energy group pairs
energygrp-table          =
; Spacing for the PME/PPPM FFT grid
fourierspacing           = 0.12
; FFT grid size, when a value is 0 fourierspacing will be used
fourier-nx               = 0
fourier-ny               = 0
fourier-nz               = 0
; EWALD/PME/PPPM parameters
pme-order                = 4
ewald-rtol               = 1e-05
ewald-rtol-lj            = 0.001
lj-pme-comb-rule         = Geometric
ewald-geometry           = 3d
epsilon-surface          = 0
implicit-solvent         = no
; OPTIONS FOR WEAK COUPLING ALGORITHMS
; Temperature coupling
tcoupl                   = Nose-Hoover ;Berendsen
; Groups to couple separately
tc-grps                  = System
; Time constant (ps) and reference temperature (K)
tau-t                    = 0.4
ref-t                    = 298.15
; pressure coupling
pcoupl                   = Berendsen ;Parrinello-Rahman ;Berendsen
pcoupltype               = Isotropic
; Time constant (ps), compressibility (1/bar) and reference P (bar)
tau_p                    = 0.8
compressibility          = 4.46e-5
ref_p                    = 1.01325
; GENERATE VELOCITIES FOR STARTUP RUN
gen-vel                  = no
gen-temp                 = 300
gen-seed                 = -1
; OPTIONS FOR BONDS
constraints              = h-bonds
; Type of constraint algorithm
constraint-algorithm     = lincs
; Do not constrain the start configuration
continuation             = yes
; Use successive overrelaxation to reduce the number of shake iterations
Shake-SOR                = no
; Relative tolerance of shake
shake-tol                = 0.0001
; Highest order in the expansion of the constraint coupling matrix
lincs-order              = 4
; Number of iterations in the final step of LINCS. 1 is fine for
; normal simulations, but use 2 to conserve energy in NVE runs.
```

```
; For energy minimization with constraints it should be 4 to 8.
lincs-iter               = 1
; Lincs will write a warning to the stderr if in one step a bond
; rotates over more degrees than
lincs-warnangle          = 30
; Convert harmonic bonds to morse potentials
morse                    = no
; Non-equilibrium MD stuff
;acc-grps                =
;accelerate              =
;freezegrps              =
;freezedim               =
;cos-acceleration        = 0
;deform                  =
```

3 Molecular Structure File:

- S8: Cyrene .gro file specifies initial molecular configurations and box dimensions for the simulations. Essential for recreating simulation setups.

SI8: Cyrene .gro file

```
MKTOP
17
    1CYR     O3    1  10.865   3.837   4.108
    1CYR     C6    2  10.848   3.946   4.163
    1CYR     C3    3  10.739   4.052   4.129
    1CYR     H4    4  10.692   4.030   4.033
    1CYR     O1    5  10.640   4.058   4.220
    1CYR     C1    6  10.709   4.135   4.341
    1CYR     H1    7  10.630   4.183   4.398
    1CYR     C4    8  10.779   4.241   4.261
    1CYR     O2    9  10.799   4.186   4.128
    1CYR     H5   10  10.717   4.330   4.256
    1CYR     H6   11  10.877   4.255   4.307
    1CYR     C2   12  10.802   4.027   4.403
    1CYR     H2   13  10.736   3.947   4.435
    1CYR     H3   14  10.855   4.063   4.492
    1CYR     C5   15  10.910   3.973   4.306
    1CYR     H7   16  10.931   3.873   4.345
    1CYR     H8   17  10.995   4.039   4.288
   1.00000   1.00000   1.00000
```

4 Comparison of charge differences between models

- SI9: Summary of the partial charges on oxygen atoms (O1, O2, O3) across four different models: GROMOS, Acetal, Acetal-Like, and Ether. These charges were derived from the respective parameterizations and

highlight the variations in electronic distribution for each model, which may influence their interactions in molecular simulations.

Model	Oxygen 1 charge (O1)	Oxygen 2 charge (O2)	Oxygen 3 charge (O3)
GROMOS	-0.425	-0.440	-0.500
Acetal	-0.400	-0.400	-0.470
Acetal-Like	-0.200	-0.200	-0.470
Ether	-0.400	-0.400	-0.470

5 Results Supporting Figures:

- Extrapolated viscosity values from NEMD simulations, highlighting the smooth trendlines used to predict viscosity at zero acceleration.
- Linear scaling law fit data for critical property predictions, including slopes and regression values.

SI10: Viscosity plotted against acceleration at each individual temperature. Viscosity at 0 nm ps^{-2} was extrapolated and plotted in Figure 7

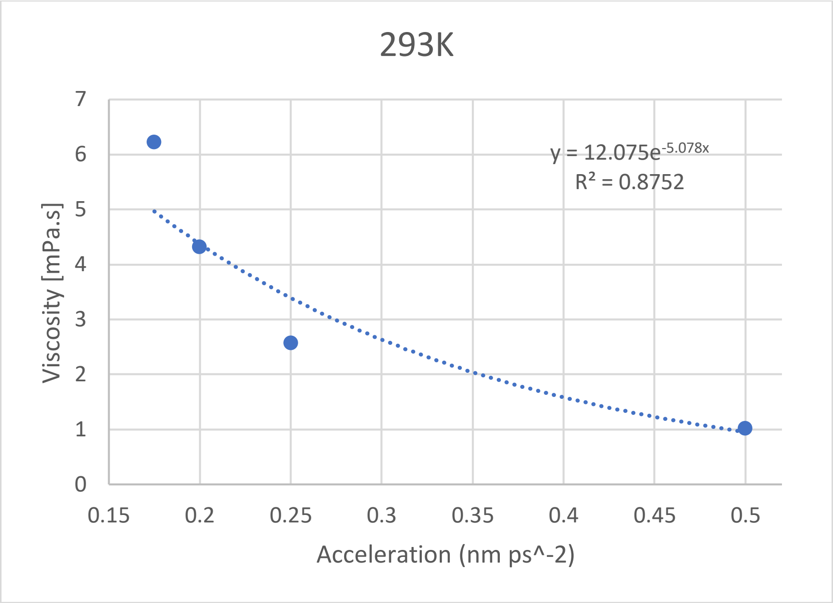
293K
y = 12.075e^-5.078x
R² = 0.8752
Viscosity [mPa.s]
Acceleration (nm ps^-2)
0
1
2
3
4
5
6
7
0.15
0.2
0.25
0.3
0.35
0.4
0.45
0.5

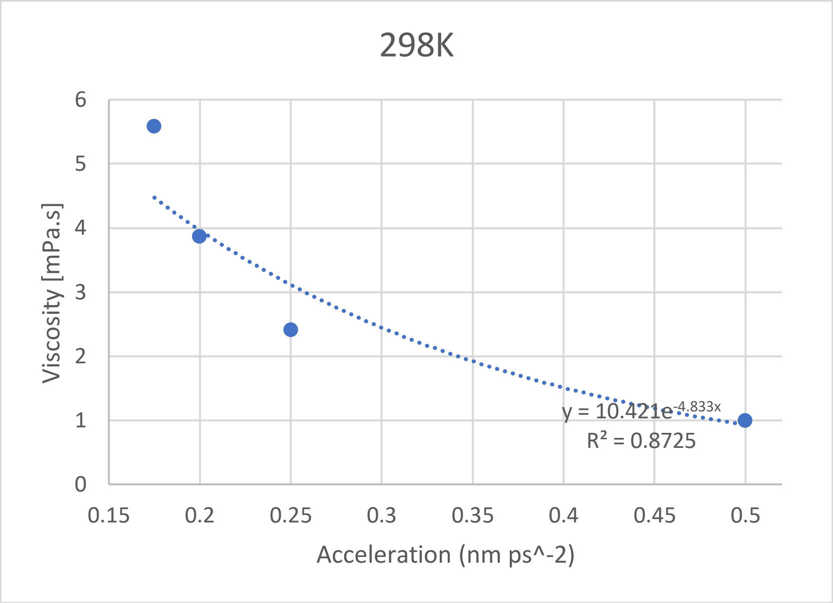
298K
y = 10.421e^-4.833x
R² = 0.8725
Viscosity [mPa.s]
Acceleration (nm ps^-2)
0
1
2
3
4
5
6
0.15
0.2
0.25
0.3
0.35
0.4
0.45
0.5

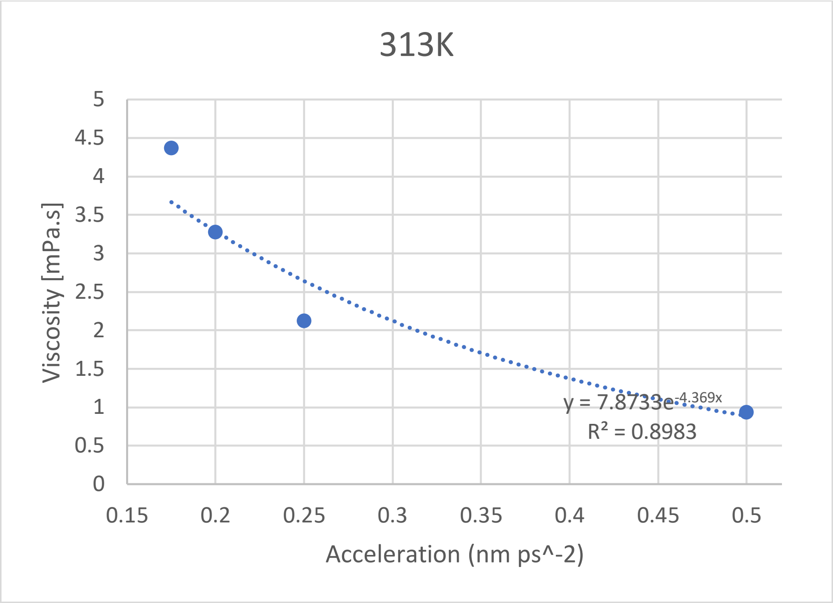
313K
Viscosity [mPa.s]
5
4.5
4
3.5
3
2.5
2
1.5
1
0.5
0
0.15
0.2
0.25
0.3
0.35
0.4
0.45
0.5
Acceleration (nm ps^-2)
y = 7.8733e^-4.369x
R² = 0.8983

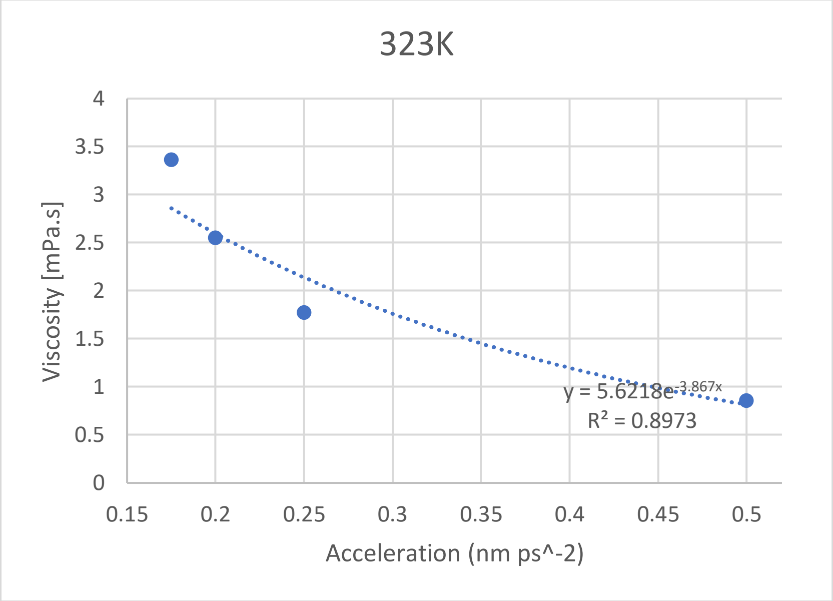
323K
Viscosity [mPa.s]
4
3.5
3
2.5
2
1.5
1
0.5
0
0.15
0.2
0.25
0.3
0.35
0.4
0.45
0.5
Acceleration (nm ps^-2)
y = 5.6218e^-3.867x
R² = 0.8973

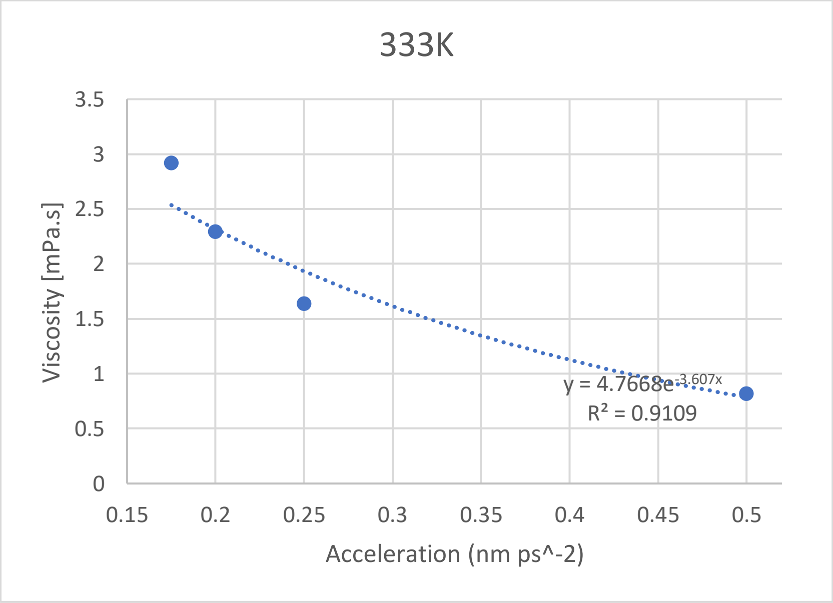
333K
Viscosity [mPa.s]
3.5
3
2.5
2
1.5
1
0.5
0
0.15
0.2
0.25
0.3
0.35
0.4
0.45
0.5
Acceleration (nm ps^-2)
y = 4.7668e^-3.607x
R² = 0.9109

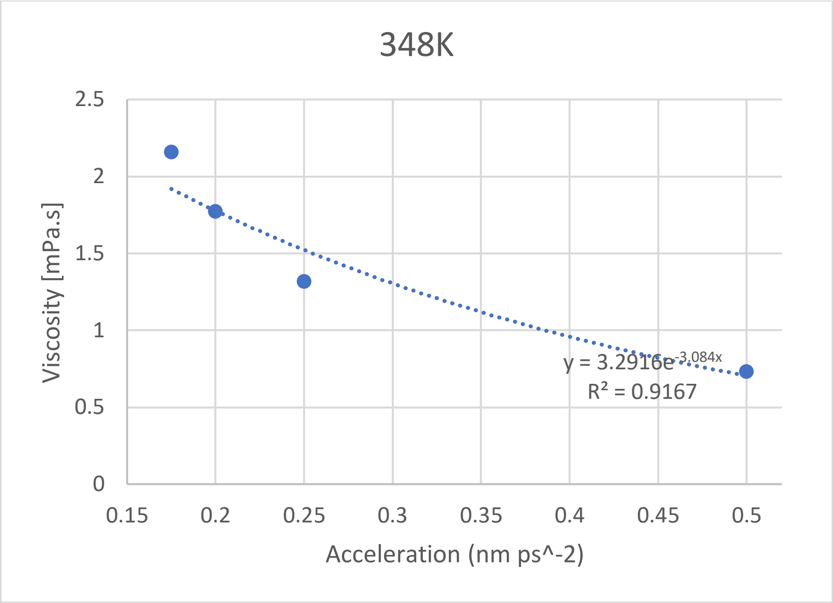
348K
Viscosity [mPa.s]
2.5
2
1.5
1
0.5
0
0.15
0.2
0.25
0.3
0.35
0.4
0.45
0.5
Acceleration (nm ps^-2)
y = 3.2916e^-3.084x
R² = 0.9167

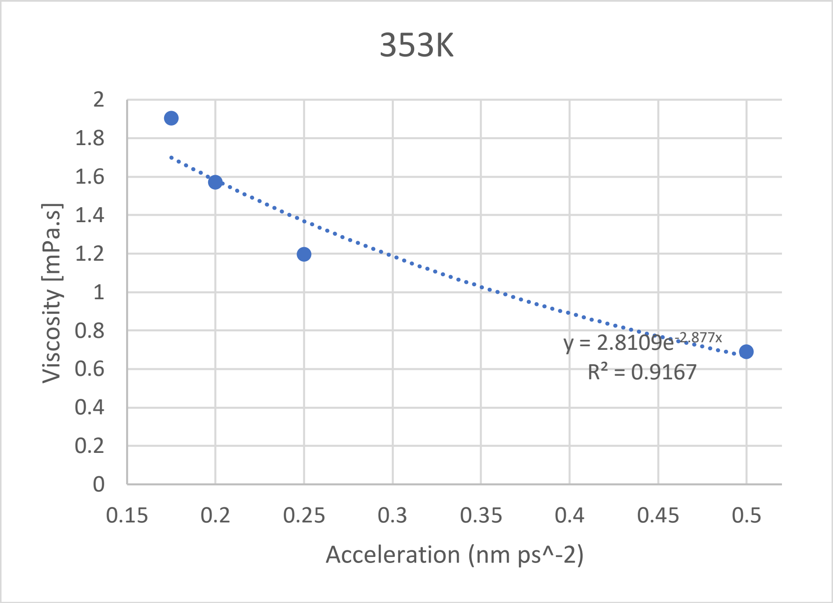
353K
Viscosity [mPa.s]
2
1.8
1.6
1.4
1.2
1
0.8
0.6
0.4
0.2
0
0.15
0.2
0.25
0.3
0.35
0.4
0.45
0.5
Acceleration (nm ps^-2)
y = 2.8109e^-2.877x
R² = 0.9167

SI11: Scaling law fit for critical property prediction

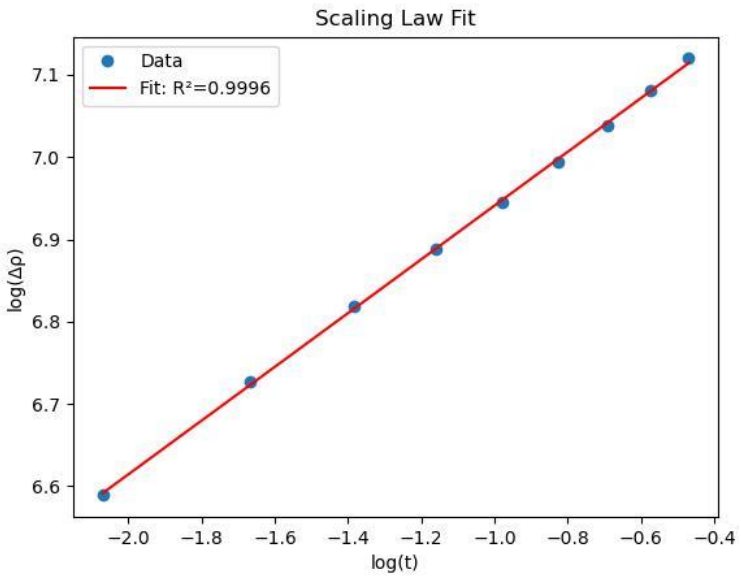

6 Dipole Moment Calculation:

- S12: Detailed step-by-step computation of the dipole moment based on atomic positions (from .gro files) and charges (from .itp files). Includes equations for vector and magnitude calculations.

SI12: Dipole moment calculation

Coordinates (x,y,z) are obtained for each model from the .gro file (S8) – coordinates are in nm and need to be converted to m ($*10^{-9}$)

Charges are obtained from each .itp file (S1-S4) – charges are in elementary charge units (e) and need to be converted to q ($*1x10^{-19}$)

For each atom i it contributes to the dipole moment by

$$\boldsymbol{p}_i = q_i \cdot r_i$$

q_i is the charge and r_i (x_i, y_i, z_i) is the position of the atom

The contribution from each atom is summed up

$$\boldsymbol{p} = \sum_{\boldsymbol{i}} \boldsymbol{p}_i = \sum_{\boldsymbol{i}} p_i \cdot r_i$$

Dipole moment vector is made up of three components:

$$p_x = \sum_i q_i \cdot x_i$$

$$p_x = \sum_i q_i \cdot y_i$$

$$p_x = \sum_i q_i \cdot z_i$$

The total dipole moment is the vector sum of the components

$$\boldsymbol{p} = (p_x, p_y, p_z)$$

and to obtain the magnitude:

$$|\boldsymbol{p}| = \sqrt{p_x^2 + p_y^2 + p_z^2}$$

SI13: Corrected diffusion for system size effects

- The predicted diffusivity values were corrected for system size effects using the methodologies outlined by Jamali et al. (2020) and Celebi et al. (2021).
- Corrections account for finite-size artifacts in molecular dynamics simulations
- $D_{sim} + \frac{k_B T}{6\pi\eta L}$
- Kb -> Boltzmann / T -> Temperature / η -> solvent viscosity / L -> box length

Temperature	Diffusion coefficient (m^2/s)	Viscosity (mPa.s)	Length (nm)	Corrected diffusion (m^2/s)
293.15	4.453E-07	12.08	4.39340	4.45304E-07
298.15	5.931E-07	10.42	4.40030	5.93105E-07
323.15	0.00000249	5.62	4.41745	2.49001E-06
348.15	0.00000654	3.29	4.45390	6.54002E-06
373.15	0.00001671	n/a	n/a	
398.15	0.00003083	n/a	n/a	
423.15	0.00005124	n/a	n/a	

- Adjustments were made for four specific temperatures where both viscosity and diffusivity data were available
- The changes in diffusivity values were minimal, with differences observed only within the fifth decimal place, indicating that the system size effects had a negligible impact on the overall trends (mention expected similar for other 3 diffusion coefficients if viscosities were done for those temperatures too)
